# Geographic Access Modeling of Emergency Obstetric and Neonatal Care in Kigoma Region, Tanzania: Transportation Schemes and Programmatic Implications

**DOI:** 10.9745/GHSP-D-17-00110

**Published:** 2017-09-27

**Authors:** Yi No Chen, Michelle M Schmitz, Florina Serbanescu, Michelle M Dynes, Godson Maro, Michael R Kramer

**Affiliations:** a Division of Reproductive Health, U.S. Centers for Disease Control and Prevention, Atlanta, GA, USA.; b Bloomberg Philanthropies, Kigoma, United Republic of Tanzania.; c Department of Epidemiology, Rollins School of Public Health, Emory University, Atlanta, GA, USA.

## Abstract

**Background:**

Access to transportation is vital to reducing the travel time to emergency obstetric and neonatal care (EmONC) for managing complications and preventing adverse maternal and neonatal outcomes. This study examines the distribution of travel times to EmONC in Kigoma Region, Tanzania, using various transportation schemes, to estimate the proportion of live births (a proxy indicator of women needing delivery care) with poor geographic access to EmONC services.

**Methods:**

The 2014 Reproductive Health Survey of Kigoma Region identified 4 primary means of transportation used to travel to health facilities: walking, cycling, motorcycle, and 4-wheeled motor vehicle. A raster-based travel time model was used to map the 2-hour travel time catchment for each mode of transportation. Live birth density distributions were aggregated by travel time catchments, and by administrative council, to estimate the proportion of births with poor access.

**Results:**

Of all live births in Kigoma Region, 13% occurred in areas where women can reach EmONC facilities within 2 hours on foot, 33% in areas that can be reached within 2 hours only by motorized vehicles, and 32% where it is impossible to reach EmONC facilities within 2 hours. Over 50% of births in 3 of the 8 administrative councils had poor estimated access. In half the councils, births with poor access could be reduced to no higher than 12% if all female residents had access to motorized vehicles.

**Conclusion:**

Significant differences in geographic access to EmONC in Kigoma Region, Tanzania, were observed both by location and by primary transportation type. As most of the population may only have good EmONC access when using mechanized or motorized vehicles, bicycles and motorcycles should be incorporated into the health transportation strategy. Collaboration between private transportation sectors and obstetric service providers could improve access to EmONC services among most populations. In areas where residents may not access EmONC facilities within 2 hours regardless of the type of transportation used, upgrading EmONC capacity among nearby non-EmONC facilities may be required to improve accessibility.

## Abstract (precis)

32% of estimated live births in the region may not be able to reach emergency obstetric and neonatal care (EmONC) services within 2 hours in dry season, regardless of the type of transportation available. However, bicycles, motorcycles, and cars provide a significant increase in geographic accessibility in some areas. Achieving good access may require upgrading non-EmONC facilities to EmONC facilities in some districts while incorporating bicycles and motorcycles into the health transportation strategy in others.

## INTRODUCTION

Poor geographic access to emergency obstetric and neonatal care (EmONC) often contributes to delays in women with obstetric complications receiving care.^1^ Such delays can result in adverse maternal and neonatal outcomes.^2^ The greatest risk of adverse health outcomes for pregnant women, unborn babies, and neonates occurs between 37 weeks of gestation (i.e., full-term pregnancies) and 7 days after delivery. While medical urgency varies with the type of obstetric complication, optimal access to EmONC facilities is usually considered to be within 2 hours of travel time, to provide lifesaving interventions for complications due to obstetric hemorrhage that require the most urgent care.^3^

Therefore, programs should strive to improve EmONC access by limiting the time required for a pregnant woman to reach an EmONC facility to within 2 hours of the onset of an obstetric complication, so that patients with the most urgent complications receive timely medical attention.^3^ Since access to adequate transportation is essential to reaching appropriate obstetric care in time, understanding both the local transportation context and how it affects EmONC service coverage may provide programs with insights on how the existing EmONC service network is being used, and how it could be used more effectively.

The Bloomberg Philanthropies-funded project, Reducing Maternal Deaths in Tanzania, has worked to improve access to basic EmONC (BEmONC) and comprehensive EmONC (CEmONC) services in the Kigoma Region of Tanzania among all hospitals, health centers, and large dispensaries providing delivery services. Due to limited road networks, poor road quality, and diverse terrain, Kigoma's population uses various forms of transportation (including 4-wheeled motor vehicles, motorcycles, boats, bicycles, and walking) to reach medical care.

In the existing body of literature, only a few studies analyzing geographic accessibility to care explored instances whereby motorcycles could be used as a primary transportation method. Similarly, very few studies have addressed the use of boat transportation in the context of health care-seeking travel, a mode of travel sometimes used for referrals of complicated cases. Transportation via boat has been well-reported in rural areas in Africa that are away from major road networks and near large bodies of water.^4^^–^^7^

In this article, we used a raster-based travel time cost surface model to examine the spatial distribution of travel time to EmONC (i.e., both BEmONC and CEmONC facilities) and the proportion of births (a surrogate measure for women needing delivery care) with good geographic access to EmONC services (i.e., within 2 hours' travel) at regional and subregional levels in Kigoma Region, Tanzania, using various transportation schemes. Because of the diverse terrain and transportation methods used, this accessibility analysis in this region may serve as a case study to explore how various means of transportation may be used to maximize access to EmONC services using an existing road network, by modeling travel time.

## METHODS

### Study Site

**FIGURE 1:**
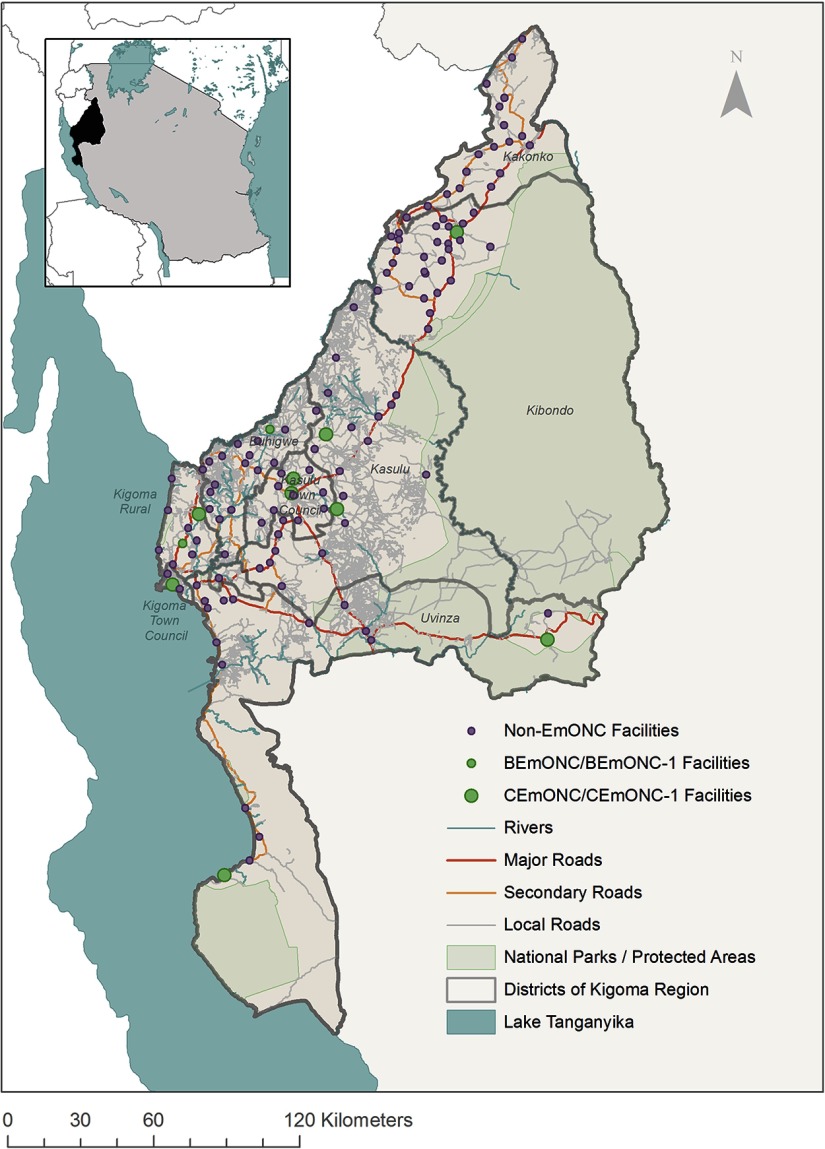
Delivery Health Facilities, by Emergency Obstetric and Neonatal Care Status, Included in the Study, Kigoma Region, Tanzania, 2013 Abbreviations: BEmONC, basic emergency obstetric and neonatal care; BEmONC-1, partially functional BeMONC facility (i.e., BEmONC without assisted vaginal delivery); CEmONC, comprehensive emergency obstetric and neonatal care; CEmONC-1, partially functional CEmONC facility (i.e., CEmONC without assisted vaginal delivery); EmONC, emergency obstetric and neonatal care.

Kigoma Region, which covers 45,066 square kilometers, is situated in the northwest corner of Tanzania and borders the Democratic Republic of the Congo, Burundi, and Lake Tanganyika (Figure 1). The region's land cover consists of grassland (34%), cropland (8%), forest (34%), and water (14%), while the remaining area consists of human settlements and other terrains. Topographically, there is a wide range of altitudes in Kigoma Region; the lowest area is 800–1,000 meters above sea level, along Lake Tanganyika, while the highest areas are in the northern and southern highlands (1,500–2,400 meters above sea level).^8^ The Luiche, Malagarasi, and Ruchuigi rivers originate from the northern and northeastern highlands and move southward before draining west into Lake Tanganyika.

In 2012, the region had a reported population of 2,127,930, of which an estimated 470,000 (22%) were women of reproductive age (i.e., 15–49 years).^9^ Kigoma Region consists of 8 administrative councils (Buhigwe, Kakonko, Kasulu, Kasulu Township Authority, Kibondo, Kigoma Rural, Kigoma Municipal-Ujiji, and Uvinza).^10^ It is characterized by its rurality (83% of surveyed households^11^), high birth rates (210 births per 1,000 women aged 15–44 years^12^), and relatively high maternal mortality (222 maternal deaths per 100,000 facility-based live births in 2013^13^). In 2014, only 47% of the deliveries in Kigoma Region were attended by a skilled birth attendant.^12^

### Accessibility Analysis Software

This analysis aims to estimate the minimum amount of time required for populations to travel to the nearest EmONC facility in Kigoma Region when seeking care, by using AccessMod version 4.0 (World Health Organization [WHO], Geneva, Switzerland), an add-on analytic extension to ArcGIS 9.3.1.^14^ This travel time modeling program uses a least-cost path (friction surface) approach to produce a raster layer across the target area, where each gridded cell represents the minimum travel time from the cell's location to the target destination. Spatial raster data models are representations of continuously varying attributes in which the surface of the earth is divided into uniformly spaced pixels of cells, and each pixel carries one or more attributes of a value for that location. Raster data differ from vector data models (e.g., points, lines, and polygons) when representing spatially continuous processes and interactions such as distance, terrain, and travel time.

This analysis aims to estimate the amount of time required for populations to travel to the nearest EmONC facility in Kigoma Region.

The travel time model requires the following data inputs: (1) geographic coordinates of the health facilities providing EmONC services; (2) combined land cover raster dataset; (3) digital elevation model (DEM) raster dataset; and (4) travel speed specification, based on land cover classification and transportation modes. Tobler's function, which corrects walking speed based on the direction of slopes on the terrain, was used to adjust the anisotropic (directional) walking speed.^15^ Motorized transportation did not require directional speed adjustments. Unfortunately, due to the limitations of our software installation, we were unable to adjust for anisotropic changes in bicycling speed due to a reported bug in AccessMod version 4.0.

### Input Data

Table 1 summarizes the data source, the spatial resolution, and other specification details of the input geospatial data used for modeling the travel time and estimating accessibility to existing EmONC services in the region. All input geospatial datasets were cropped to the administrative boundaries of Kigoma Region, as derived from the ward boundaries of the 2012 Tanzania census.^10^ All rasters used in the travel time analysis were specifically at 30-meters resolution, while the live birth raster data used in the live births analysis were kept at 100-meters resolution, as it was recommended not to resample that dataset. All data layers were projected into the spatial reference frame, WGS84/UTM Zone 35S.

**TABLE 1.** Characteristics of the Input Geospatial Datasets, by Data Layer, for Modeling Travel Time and Estimating Accessibility Coverage Among Women Needing Delivery Care in Kigoma Region, Tanzania

	Data Layer					
	Land Cover^a^	River Network	Road Network^b^	Digital Elevation Model	Birth Density Map^c^	Health Facility Coordinates
**Data format**	Raster	Shapefile	Shapefile	Raster	Raster	Shapefile
**Year**	2010	2015	2015	2000	2012	2016
**Source**	Regional Centre for Mapping of Resources for Development, NASA SERVIR Global^21^	OpenStreetMap	OpenStreetMap	Shuttle Radar Topography Mission^22^	WorldPop^29^	CDC Kigoma Health Facility Assessment^16^
**Spatial resolution**	30 meter	N/A	N/A	30 meter	100 meter	N/A
**Purpose**	Provide non-road land feature class (i.e., forestland, grassland, cropland, settlement, wetland)	Provide a layer of physical barrier in addition to wetland	Provide road land feature class (i.e., major roads, major roads crossing residential areas, secondary roads, local roads)	Provide elevation/slope landscape used for travel speed adjustment for walking	Provide estimated distribution count of live births for a specified travel time catchment	Provide locations of assessed coordinates in the analysis

^a^ The 2010 Tanzania land cover scheme I was used to specify non-road land classes.

^b^ Roads are reclassified to reflect the classification scheme used by John Snow, Inc. and Medical Supply Department in the cross-country medical supply route analysis in 2014.^18^

^c^ The layer was created using the mapping methodology described by Tatem et al. (2014).^46^

#### Health Facility Dataset

The health facility dataset was derived from a 2013 region-wide health facility assessment led by the U.S. Centers for Disease Control and Prevention (CDC) staff in selected Kigoma Region health facilities to document the functionality of EmONC infrastructure and EmONC-related human resources.^16^ During the same time frame, the CDC and project partners used the Pregnancy Outcomes Mortality Surveillance (POMS) system to document changes in facility-based deliveries in the region and to assess corresponding pregnancy outcomes in the region.^13^ Together, these 2 surveys documented evidence for the observed practice of the 9 essential medical services necessary for treating and managing maternal and neonatal complications (referred to as signal functions^17^), both at the facility level (through the health facility assessment) and at the individual delivery level (through POMS).

Included in this study were 127 health facilities in the region (Figure 1), composed of hospitals, health centers, and large dispensaries providing delivery services (i.e., dispensaries experiencing more than 90 births per year), accounting for 97% of all facility deliveries in 2012 in Kigoma Region. A total of 11 facilities were found to be providing EmONC levels of care. Eight facilities (4 hospitals and 4 health centers) were identified as fully functional CEmONC facilities, performing 9 signal functions for EmONC within the 3 months before the assessment; 1 facility was identified as a partially functional CEmONC facility (CEmONC-1), performing 8 signal functions excluding the provision of assisted vaginal delivery (AVD).^16^ Two additional health centers were found to be partially functional BEmONC facilities (BEmONC-1), defined as performing 6 BEmONC signal functions, excluding AVD. Despite being unable to provide AVD, the 3 partially functional BEmONC or CEmONC facilities were found to have strong enough transportation referral networks to ensure their inclusion in the roster of EmONC facilities. The geographic coordinates used to locate the facilities in this study were recorded in the health facility assessment, using Garmin eTrex 30 devices with an accuracy of 3–5 meters.

#### Combined Land Cover Dataset

A road network dataset, obtained from OpenStreetMap, was reclassified to reflect the classification scheme used by John Snow, Inc. (JSI) and the Medical Supply Department (MSD) of Tanzania for their 2014 cross-country medical supply route analysis.^18^ The road classes included: (1) major roads; (2) major roads crossing residential areas; (3) secondary roads; and (4) local roads^18^ (road network visualized in Figure 1). Local roads were further divided into categories, based on road width and OpenStreetMap classifications, including the following: (1) car-passable roads; (2) motorcycle- and bicycle-passable roads (i.e., tracks that are passable to motorcycle and bicycle, but not cars); and (3) walking-only roads.^19^^,^^20^ Boating routes were digitized to allow for travel approximately 60 meters away from the shore of Lake Tanganyika. Docks were identified using Bing satellite imagery and were digitized to connect to boat travel routes.

Both river and road network vector datasets, obtained from OpenStreetMap, were transformed to raster datasets consisting of 30-meter gridded cells, and were then overlaid on the land cover raster dataset.^21^ This created a combined land cover raster dataset with 13 unique land feature classes. The 6 non-road land cover classes included forest land, grassland, cropland, settlements, wetlands, and other land. For the purposes of analysis, wetlands and rivers were considered to be impassable to any form of transportation.

#### Digital Elevation Model

To provide a mapped model of land elevation, we obtained Shuttle Radar Topography Mission (SRTM) digital elevation model data, at a spatial resolution of 30 meters (1 arc-second), from the U.S. Geological Survey.^22^

#### Travel Scenarios

To simulate the use of various primary transportation modes in real life, we specified 4 different travel scenarios according to the primary transportation modes (i.e., walking, bicycle, motorcycle, or car) described in the 2014 Kigoma Reproductive Survey (RHS) conducted by the CDC:

1. Walking Scenario (Scenario 1): walking and boat
2. Cycling Scenario (Scenario 2): walking, bicycling, and boat
3. Motorcycle Scenario (Scenario 3): walking, motorcycle taxi, and boat
4. Car Scenario (Scenario 4): walking, 4-wheeled motor vehicle, and boat

We specified 4 different travel scenarios according to whether the primary transportation mode was by walking, bicycle, motorcycle, or car.

Boats were included for every scenario, as there are many villages around the lake that may use boats for at least part of the trip. A boat route was included as part of a journey if its inclusion resulted in an overall shorter travel time. Walking was also included in every scenario, because all residents would need to walk at some point during their trip to an EmONC facility, as they travel through land areas with low road coverage.

To simulate real-life travel experiences in which travel time may vary by terrains, road types, and transportation used, our travel time computation module specified a transportation mode with a corresponding travel speed for every combined land cover class, under each travel scenario. Various sources were used to ascertain the transportation-specific travel speed for each land cover type in the dry season, as summarized in Table 2; the sources consisted of the Global Accessibility Map,^23^ AccessMod version 3.0 publication literature,^24^ a cost-surface analysis conducted in Dar es Salaam,^25^ WHO's Tanzania road safety brief,^26^ a motorcycle analysis conducted in Hanoi, Vietnam,^27^ and a cost-distance analysis conducted in the Biliran Island, Philippines.^28^ Table 3 describes the travel speed for each land cover class for all 4 travel scenarios employed in this analysis.

**TABLE 2.** Source and Rationale for Travel Speed Specification per Land Cover Type in Kigoma Region, Tanzania

Land Cover Type	Walking	Bicycling	Motorcycle	4-Wheeled Motor Vehicle	Boat
Forestland	Global Accessibility Map (“tree cover/broadleaved and tree cover/mixed leaf type”)	N/A	N/A	N/A	N/A
Grassland	Global Accessibility Map (“shrub cover, close-open, evergreen/deciduous, mosaic: cropland/shrub/grass cover, and cultivated and managed area”)	AccessMod version 3.0 publication literature^24^ (cycling on “low-density vegetation”)	AccessMod version 3.0 publication literature^24^ (cycling on “low dense vegetation”) for simulating reduced motorcycling speed on non-road land features	N/A	N/A
Cropland	Same as in the cell above	N/A	N/A	N/A	N/A
Settlement	Half of the urban walking speed (5 km/hr) specified in AccessMod version 4.0 user manual to approximate the reduced speed of a pregnant woman walking or being carried on a stretcher	AccessMod version 3.0 publication literature^24^ (cycling on “built areas”)	AccessMod version 3.0 publication literature^24^ (cycling on “built areas”) for simulating reduced motorcycle speed on non-road land features	N/A	N/A
Other land classification	Global Accessibility Map (“bare area”)	AccessMod version 3.0 publication literature^24^ (cycling on “low-density vegetation”)	AccessMod version 3.0 publication literature^24^ (cycling on “low-density vegetation”) for simulating reduced motorcycling speed on non-road land features	N/A	N/A
Boat route	N/A	N/A	N/A	N/A	Local knowledge
Major roads	Global Accessibility Map (“bare area”)	AccessMod version 3.0 publication literature^24^ (cycling on “main road”)	The average motorcycling speed per road class recorded from studies done in Vietnam and the Philippines.	Average speed data taken either from WHO's United Republic of Tanzania Road Safety Brief or from a time-distance study done in Dar es Salaam.	N/A
Major roads crossing residential areas					N/A
Secondary roads					N/A
Local roads: passable to all transportation			Set to be equal to 4-wheeled motor vehicles, under the local assumption that motorcycles usually cannot travel faster than these vehicles on roads		N/A
Local roads: passable to motorcycle/bicycle				N/A	N/A
Local roads: walking only		N/A	N/A	N/A	N/A

Summary of the original speed data sources and rationales from which the traveling speeds were derived per transportation and landcover type, for constructing a traveling scenario table required by AccessMod version 4.0 analysis modules to estimate the travel time and accessibility to existing emergency obstetric and neonatal care services in Kigoma Region.

**TABLE 3.** Travel Speeds, per Land Cover Type, to the Nearest Emergency Obstetric and Neonatal Care Facilities in Kigoma Region, Tanzania, by Travel Scenario

Land Cover Type	Travel Speeds (km/hr)													
	Walking Scenario (Scenario 1)		Cycling Scenario (Scenario 2)			Motorcycle Scenario (Scenario 3)			Car Scenario (Scenario 4)		
	*Walking*^a^	*Boat*	*Walking*	*Bicycling*^*b*^	*Boat*	*Walking*	*Motorcycle Taxi*	*Boat*	*Walking*	*Car*	*Boat*
Forestland	1.0	–	1.0	–	–	1.0	–	–	1.0	–	–
Grassland	1.7	–	–	7.0	–	–	7.0	–	1.7	–	–
Cropland	1.7	–	1.7	–	–	1.7	–	–	1.7	–	–
Settlement	2.5	–	–	7.0	–	–	7.0	–	2.5	–	–
Other land cover	2.5	–	–	7.0	–	–	7.0	–	2.5	–	–
Boat route	–	15.0	–	–	15.0	–	–	15.0	–	–	15.0
Major roads	2.5	–	–	10.0	–	–	40.2	–	–	50.0	–
Major roads crossing residential areas	2.5	–	–	10.0	–	–	26.2	–	–	30.0	–
Secondary roads	2.5	–	–	10.0	–	–	35.2	–	–	40.0	–
Local roads: passable to all transportation	2.5	–	–	10.0	–	–	15.0	–	–	15.0	–
Local roads: passable to motorcycle/bicycle	2.5	–	–	10.0	–	–	15.0	–	2.5	–	–
Local roads: walking only	2.5	–	2.5	–	–	2.5	–	–	2.5	–	–

^a^ Tobler's function was used for correcting anisotropic movement.

Wetlands and rivers were considered to be impassable for the purposes of this analysis (i.e., walking speed of 0), and were not included in this table.

#### Live Birth Density Dataset

Live birth count was used as a proxy measurement for women needing delivery care, which is the target population of EmONC services. A 2012 projected live birth raster dataset for Tanzania was obtained from the WorldPop Project.^29^ The value of each 100-meter gridded cell represented the estimated number of live births that would have occurred in an area of 100 square meters in 2012.

### Analysis

A raster layer that describes the minimum travel time required to reach the nearest EmONC facility was created using the AccessMod version 4.0 extension's modules for each of the 4 travel scenarios. For this accessibility analysis, the upper limit of the estimated travel time was set at 2 hours, a conservative time frame consistent with the WHO recommendations for access to EmONC facilities.^30^ Therefore, “good geographic access to EmONC care” was operationally defined as a woman's travel time to EmONC care being at, or under, 2 hours, while “poor geographic access to EmONC care” was defined as a woman's travel time to EmONC care exceeding 2 hours.

Each scenario-specific travel time raster layer was reclassified and converted into 4 incremental 30-minute travel time zones (up to 2 hours) as polygon vectors in ArcGIS 10.3. All 2-hour service catchment areas for each corresponding travel scenario were merged to show the distribution of areas with good EmONC service access (i.e., areas within which one can reach EmONC services in less than 2 hours) based on each of the primary transportation modes.

To compute the region-wide proportion of live births in a travel time zone or service catchment, we divided the total number of live births within a travel time zone or service catchment by the total number of live births in Kigoma Region. In addition, the proportion of live births with poor access to EmONC under each travel scenario was calculated per administrative council for each travel scenario (i.e., Scenarios 1–4 and the “all-modes” scenario, where women may use any of the 4 travel scenarios to reach the nearest EmONC facility as necessary), by dividing the total number of live births located outside the 2-hour service catchment in an administrative council by the total number of live births in the entire council. The live birth figures involved in these calculations were aggregated from the 2012 live birth raster dataset in ArcGIS 10.3, collected to a 100-meter resolution by various travel time or 2-hour catchment polygon vectors using zonal statistics.

The estimate for the number of all births per catchment presented in this analysis was the product of the total population of women aged 15–49 years in Kigoma Region in 2012,^9^ the annual population growth coefficient (for projecting the population of 2013),^9^ the age-specific fertility rate in the 2014 Kigoma RHS,^12^ and the proportion of births occurring in that catchment.

### Ethical Considerations

This study was reviewed and approved by the CDC's Center for Global Health Human Subject Review Board and was determined not to comprise human subjects research.

## RESULTS

### Geographic Access to the Nearest EmONC Facility by Transportation Mode

**FIGURE 2:**
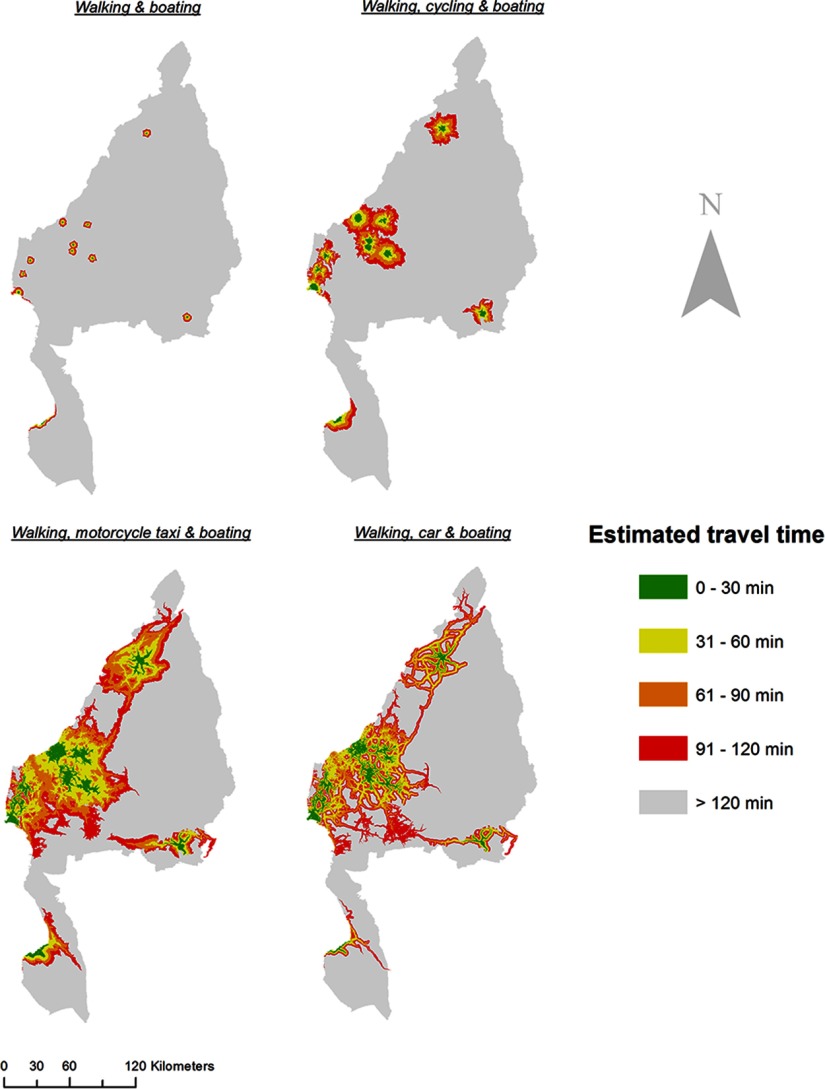
Distribution of Estimated Travel Time to the Nearest Emergency Obstetric and Neonatal Care Facility by Travel Scenario, Kigoma Region, Tanzania

Compared with the walking scenario (Scenario 1), in which the travel distance covered within 2 hours was confined to the immediate surroundings of each EmONC facility, the travel distance greatly increased once people could access bicycles (Scenario 2), motorcycles (Scenario 3), or cars (Scenario 4) (Figure 2). The greatly improved accessibility along the road network was not visually evident until people traveled by motor vehicles (Scenarios 3 and 4) (Figure 2). The travel time distributions by motorcycle (Scenario 3) and by bicycle (Scenario 2) was less linked to the road network than was traveling by car, as bicycle and motorcycle taxis were assumed to be capable of traveling through the grassland and other land cover surrounding many roads.

Many towns located near the shore of Lake Tanganyika—particularly those in the southwest—were connected to some levels of a road network (Figure 1) and were mostly within the 2-hour travel time catchment from an EmONC facility if motorized vehicles were used (Figure 2). A few areas on the northern shore were not connected to any EmONC facilities by the road network but were able to gain marginal EmONC access (e.g., travel time of 91–120 minutes) in scenarios in which motorized vehicles were used, since boat routes provided connections to the closest road network.

**FIGURE 3:**
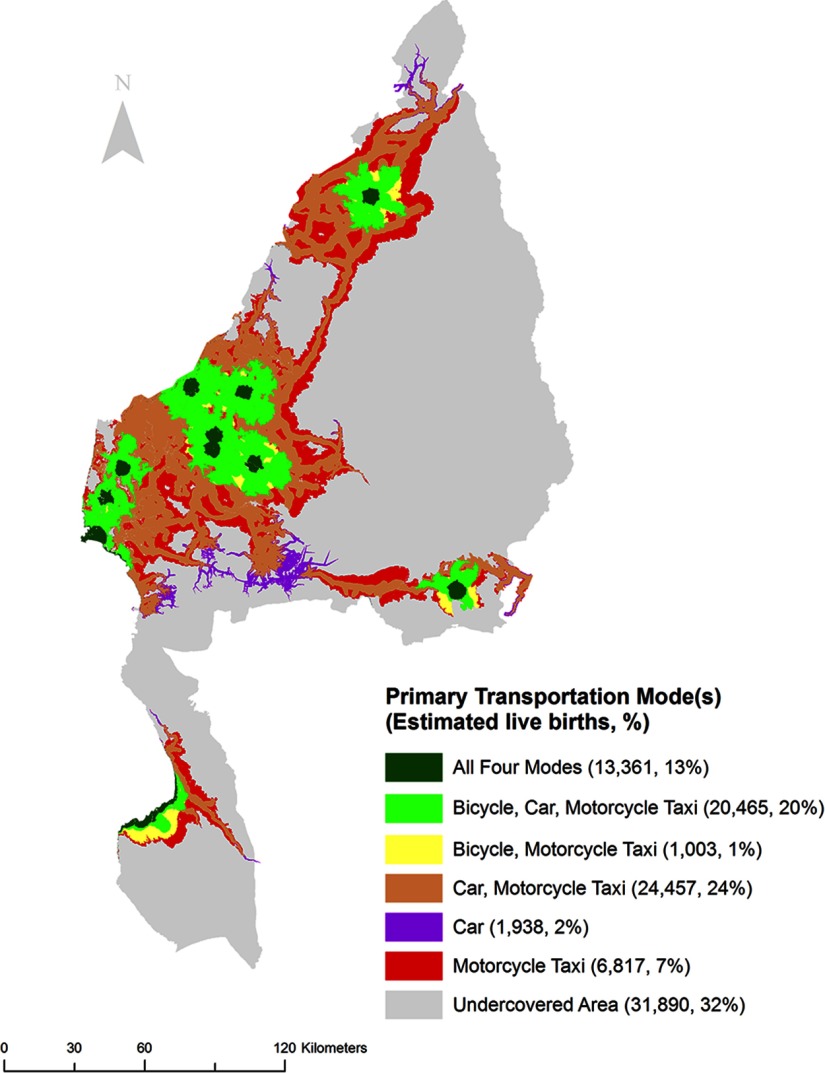
Primary Transportation Modes Allowing Access to the Nearest Emergency Obstetric and Neonatal Care Facility Within 2 Hours, Kigoma Region, Tanzania

The region-wide percentage of live births with poor access to EmONC decreased with faster methods of transportation, with the walking scenario (Scenario 1) having the highest percentage of poor access (87%), followed by mechanized transportation (i.e., Scenario 2: 65%), and finally motorized transportation (i.e., Scenario 4: 39%; Scenario 3: 33%) (Table 4). Figure 3 displays the areal distribution of Kigoma Region by primary transportation modes (travel scenarios) that women could use to reach EmONC within 2 hours. In other words, each colored catchment area displays all the primary transportation modes, or travel scenarios (of the 4 in this analysis), that women in that area may use to reach the nearest EmONC facility within 2 hours. Even in the ideal scenario where people may use any of the 4 primary transportation modes, about one-third (32%) of the estimated live births would not reach EmONC facilities within 2 hours in Kigoma Region. One-third of live births (i.e., 24% [Car, Motorcycle Taxi] + 2% [Car] + 7% [Motorcycle Taxi] = 33%) occurred in areas where the population could reach an EmONC facility only if motorized vehicles (i.e., motorcycle [Scenario 3] or car [Scenario 4]) were available. Seven percent of the live births occurred in areas where the population may only reach EmONC services if a motorcycle taxi (Scenario 3) were available.

**TABLE 4.** Distribution of Estimated Proportion of Live Births Occurring in Each Travel Time Catchment by Travel Scenario,^a^ Kigoma Region, Tanzania, 2013

Travel Time (min)	Walking Scenario (Scenario 1) No. (%)	Cycling Scenario (Scenario 2) No. (%)	Motorcycle Scenario (Scenario 3) No. (%)	Car Scenario (Scenario 4) No. (%)
0–30	1,263 (2)	9,044 (11)	17,418 (21)	16,572 (20)
31–60	3,172 (4)	7,542 (9)	16,487 (20)	13,565 (16)
61–90	3,451 (4)	6,044 (7)	13,320 (16)	11,768 (14)
91–120	3,123 (4)	6,522 (8)	8,001 (10)	8,631 (10)
>120	71,980 (87)	53,837 (65)	27,763 (33)	32,453 (39)

^a^ Walking scenario (Scenario 1) includes both walking and boat access. Cycling scenario (Scenario 2) includes walking, boat access, and bicycle access. Motorcycle scenario (Scenario 3) includes walking, boat access, and motorcycle access. Car scenario (Scenario 4) includes walking, boat access, and car access.

About one-third of estimated live births would not reach an EmONC facility within 2 hours in Kigoma Region in the ideal travel scenario.

### Distribution of Live Births With Poor Access to EmONC per District by Transportation Mode

**FIGURE 4:**
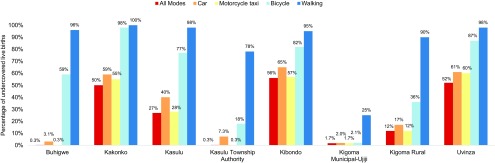
Percentage Distribution of Live Births With Poor Access to Emergency Obstetric and Neonatal Care Facilities, by District, for Each Travel Scenario in Kigoma Region, Tanzania

Among the 8 administrative councils, Kakonko, Kibondo, and Uvinza consistently were estimated to have more than half of their live births experiencing poor access to EmONC service regardless of the travel scenario used (Figure 4). Kigoma Muncipal-Ujiji was the only council where the percentage of live births with poor EmONC access remained below 30% across all travel scenarios. It was also the only council where no meaningful difference in accessibility was observed among the 3 types of vehicles (i.e., bicycle, motorcycle taxi, and cars; Scenarios 2, 3, and 4, respectively).

In the scenario where bicycles alone were used, for 3 councils (Kakonko, Kibondo, and Uvinza), more than half of the women could not get to care within 2 hours, even if they used any of the 4 primary transportation modes (all-mode scenario). Kigoma Municipal-Ujiji and Kasulu Township Authority had fewer than 20% of live births experiencing poor EmONC access when bicycles were used (Figure 4). In scenarios in which motorized vehicles were fully utilized (Scenarios 3 and 4), only half of the councils in Kigoma Region (Buhigwe, Kasulu Township Authority, Kigoma Municipal-Ujiji, and Kigoma Rural) had fewer than 20% of live births experiencing poor EmONC access.

## DISCUSSION

Our study provides a lens for public health stakeholders to focus on the disparity in geographic access to EmONC services across Kigoma Region in Tanzania. By using spatial health care accessibility modeling, stakeholders can identify locations where various interventions, such as increasing transportation access or health resources, could be implemented to effectively improve access. The fact that 32% of estimated live births in Kigoma Region may not be able to reach EmONC services within 2 hours in dry season, regardless of the type of transportation used, suggests that a transportation-based intervention alone may not be enough to achieve a high level of accessibility to EmONC services. Our results in Kakonko, Kibondo, and Uvinza suggest it may be necessary to upgrade non-EmONC facilities, especially health centers, to have EmONC capabilities.

A transportation-based intervention alone may not be enough to achieve a high level of accessibility to EmONC services in Kigoma Region, Tanzania.

We therefore recommend to implementing partners that to address coverage gaps, the priority for upgrades should be given to facilities located in areas with suboptimal estimated travel time but with a higher density of births, where the demand for delivery care is likely to be greater. Another possible programmatic intervention may include establishing affordable maternity waiting homes in the geographic proximity of CEmONC facilities such that women living farther than 2 hours away from any EmONC facilities may stay during the last weeks of their pregnancy. However, to ensure the effectiveness of such an intervention, connection to appropriate transportation from the maternity homes and promotion of using the homes via social network and community support should be established.^31^ As several hospitals and health centers in Kigoma are associated with local maternity waiting homes, future research may be required to assess the usage of the homes and their impact to EmONC access by analyzing travel time.

Our findings also suggest that there are areas where geographic access to EmONC might be enhanced by improving women's access to mechanized or motorized vehicles. Similar trends were also observed in a study that used travel time cost surface modeling to assess accessibility by various transportation scenarios in the Western Province of Rwanda.^32^ Since access to a transportation mode may depend on both affordability and availability, it is crucial to consider the advantages and disadvantages of each of the primary methods of transportation when considering health transportation strategies. Although 4-wheeled motor vehicles have the advantages of greater geographic reach, increased long-distance travel capabilities, and potential space for medical equipment, they may be a less practical option for ambulance transportation given the relatively low availability of functional vehicles, insufficient fuel, monetary cost, and lack of available drivers,^16^ especially in rural communities.

There are areas where geographic access to EmONC might be enhanced by improving women's access to mechanized or motorized vehicles.

In sub-Saharan Africa, 4-wheeled motor ambulance services are rarely available for the general population due to the high costs associated with maintaining such vehicles.^33^ Furthermore, ambulances are frequently used for other duties unrelated to patient transportation in clinical emergencies, making them even less accessible.^1^ Another limitation of 4-wheeled public transportation vehicles is that they are mostly operated around urban areas or town centers, particularly on major interurban roads.^34^^,^^35^ Therefore, unless women living in rural areas reside in villages close to major roads, it is unlikely they will be able to access 4-wheeled vehicle transportation in a timely fashion.

Many studies have stressed the importance of motorcycles and bicycles as intermediate means of transportation to improve access to health services, taking advantage of their high availability and mobility.^33^^,^^36^^–^^38^ Although these transportation methods may not be optimal for traveling over very long distances due to their susceptibility to wear and tear in rough terrain or extreme weather and higher overall taxi fares for longer distances,^34^^,^^39^ it has been suggested that they may be useful for bridging the accessibility gap in rural communities by transporting women from isolated villages to nearby traffic hubs, where women can access public transportation to reach EmONC facilities.

Our estimates of the proportion of live births with poor access by transportation method (Figure 4) suggest that the areas around Kigoma Town—Kigoma Municipal-Ujiji, Kigoma Rural, and Kasulu Township Authority—would be the most suitable districts for using bicycles for health transportation. Residents in Buhigwe, Kasulu, and Kigoma Rural districts could benefit greatly if motorcycles were successfully integrated to the local health transportation, given the large proportion of live births that would have sufficient geographic access under the motorcycle scenario. Using motorcycles in isolated areas may be especially important in Kasulu district, where there could be an expected 12% decrease in the live births with poor EmONC access if motorcycles were used, compared with the scenario in which 4-wheeled motor vehicles were used.

The areas around Kigoma Town seem to be the most suitable districts for using bicycles for health transportation, while residents in Buhigwe, Kasulu, and Kigoma Rural districts could benefit from access to motorcycles.

One major challenge to systematically incorporating motorcycles into health transportation is that the number of motorcycles in rural Tanzania is relatively low, compared with rural regions of some other African countries where motorcycles compose the majority of their transportation fleets.^35^ This may explain the relatively low use (14%) of motorcycles for traveling to advanced health facilities (e.g., hospitals and health centers) in Kigoma Region, as shown in the 2014 RHS (Supplement). Another potential factor is the cultural and social stigma against women riding on motorcycles in many sub-Saharan African regions.^40^^–^^42^ Although there has been an increasing trend in motorcycle imports in Tanzania in recent years,^34^^,^^43^ effectively incorporating motorcycles as part of routine health transportation would require continued collaboration between private and public transportation sectors and EmONC facilities, as well as promoting the use of motorcycle-based transportation intervention to rural populations.

Bicycles are one of the most common modes of transportation in rural Tanzania.^34^ However, only 6% of delivery trips to advanced health facilities involve bicycles, according to the weighted distribution of transportation type among the most recent delivery trips to health centers and hospitals in Kigoma obtained from 2014 RHS (Supplement). Furthermore, the overall utility of the bicycle as effective health transportation for women in labor may still be vastly limited by its susceptibility to wear and tear by rough road and weather conditions, which may downplay its traveling speed, as well as limited seating space and physical discomfort experienced by pregnant women during the bike ride. Consequently, bicycles may only be used in very limited travel situations, such as traveling from areas where women may not reach EmONC by stretcher (i.e., being carried on a stretcher by walking men) within 2 hours and where discomfort and tear associated with traveling distance is acceptable. A possible solution for resolving limited seating space could involve modifying bicycles to include lightweight trailers for carrying pregnant women. In-depth multidisciplinary assessments that consider transportation management, cost-effectiveness, and sociocultural factors will be required to effectively strategize the integration of bicycles and motorcycles into local health transportation systems in Kigoma Region.

Finally, our findings suggest that boat rides may provide women living in geographically secluded villages along the northeastern shore of Lake Tanganyika with indispensable access to nearby transportation hubs, where they may subsequently reach EmONC facilities via local road networks. Therefore, it may be crucial to develop multistakeholder partnerships among local community financing programs, EmONC facilities, boat ferries operators, and public transportation to facilitate affordability and a smooth transportation network transition. However, boat ferries have the disadvantage of being subject to adverse weather and poor safety regulations.^44^ Therefore, a long-term solution to enhancing EmONC access in these remote areas may still involve expanding local obstetric health resources, including upgrading the nearest health centers.

Boat rides may provide women in geographically secluded villages along the northeastern shore with indispensable access to nearby transportation hubs.

### Limitations

A major caveat for this analysis, as well as for other studies using travel time cost surface models, is that a single idealized traveling situation was assumed for each primary transportation scheme. Our schema assumed that people will access (and only use) the prespecified primary transportation on the nearest road until they reach the closest road to the nearest EmONC facility. Furthermore, all of the accessibility maps were modeled based on the road network distribution and vehicular traveling speeds for the dry season, which may not account for the potential barriers caused by flooding or poor road conditions during the rainy season. Therefore, caution should be applied when attempting to generalize such accessibility maps to real-life scenarios in which motor vehicle access may be limited based on one's residence, financial capacity, current road conditions, and local climate. In addition, the technical limitation observed when modeling anisotropic cycling speed may lead to overestimation of travel time in areas where women are traveling downhill, or underestimation of travel time in areas where women are traveling uphill.

Many of our input data layers, while collected around similar time periods (as shown in Figure 1), are not from exactly the same time period. The digital elevation model (collected in 2000) and land cover (collected in 2010) stayed relatively static in the study area throughout the time period. Meanwhile, the data layers more subject to change were aligned to match with the approximate time period of our study (i.e., the road and river networks collected in 2015, birth density map collected in 2012). While it is very unlikely that these layers are meaningfully different from their distribution in 2013, we recognize the minor impact that this data collection time discrepancy may potentially have on the accuracy of our accessibility simulation.

With regard to the underlying data used, crowdsourced road data such as OpenStreetMap are inevitably subject to inconsistent data quality, as quality depends on mappers' experience, despite numerous quality assurance efforts.^45^ The number of digitized roads saved in the database for a specified area may depend on the availability of active OpenStreetMap projects in that area, as online contributors are more likely to digitize features requested by active OpenStreetMap projects. This can potentially lead to us underestimating road access in certain areas in Kigoma Region. In addition, there is little evidence on the walking speed for people carrying a woman on a stretcher (a common method of travel for pregnant women in labor in sub-Saharan Africa), or the travel speed of motorcycle by road type in rural sub-Saharan Africa. Lack of this evidence may limit the accuracy of the travel time catchment area and the proportion of covered live births reflected in this study. Furthermore, the speeds used in the analysis may not account for geographic differences or traffic flows between originating locations and Kigoma Region. There may be overestimation of travel time in scenarios where 4-wheeled motor vehicles were used, as Dar es Salaam, the source location for the 4-wheeled motor vehicle travel speeds, may have heavier traffic than Kigoma. Conversely, there may be underestimation in scenarios where motorcycles were used, as motorcycles tend to travel much faster on paved roads, which may be more prevalent in Hanoi, one of the source locations for the motorcycle speeds, than in Kigoma.

In this analysis, geographic considerations have to be made to the underlying spatial resolution of the raster datasets used and in the aggregation of the proportion of live births with poor access to EmONC care. Any output travel time raster is affected by the quality of the spatial resolution of the input datasets. As road network is rasterized from vector data, the travel speed for a specific road class is applied to the whole 30-meter square cell, even though many roads are below a width of 30 meters. This can lead to overestimation of access in areas around the roads. In addition, there are inherently greater uncertainties about subnational data within WorldPop's birth density map.^46^ This can affect the accuracy of the estimated proportion of live births with poor EmONC access reported in areas where local population densities are significantly lower (e.g., Kibondo administrative council) than the overall average. The travel time zone and 2-hour catchment raster were both converted to polygon vectors before aggregating birth estimates via zonal statistics. Despite the high resolution (i.e., 30 meters) of the travel time friction surface, such conversion may affect the accuracy of the birth estimates around the travel time zone or 2-hour catchment border. Similarly, our inability to resample birth grids to finer resolution, due to a lack of complete and accurate high-resolution spatial data on housing distribution, may also affect the accuracy of our birth estimates, especially for smaller-sized travel time zones or 2-hour catchment areas.

## CONCLUSION

Bicycles, motorcycles, and cars provide a significant increase in geographic accessibility to EmONC services in Kigoma Region, Tanzania, but the utility of each primary transportation method may vary locally. Therefore, in order to develop an effective yet feasible health transportation intervention, stakeholders should carefully consider the capacity of their current resources while collaborating with health facilities and public transportation sectors to incorporate bicycles and motorcycles as part of the local health transportation routine. In areas where motorized transportation did not achieve satisfactory improvement to geographic accessibility, upgrading EmONC capacity among local dispensaries and non-EmONC health centers while improving health transportation should maximize local geographic access to EmONC services. Future directions of research, pending availability of other forms of data, could include rainy/dry season-specific analyses, as well as travel-time analyses to maternity homes.

Bicycles, motorcycles, and cars significantly increase geographic accessibility to EmONC services in Kigoma Region, but the utility of each primary transportation method may vary locally.

## Notes

**Peer Reviewed**

**First Published Online:** 2017 August 8
